# Inhibition of the epigenetically activated miR-483-5p/IGF-2 pathway results in rapid loss of meningioma tumor cell viability

**DOI:** 10.1007/s11060-023-04264-z

**Published:** 2023-02-21

**Authors:** Erik J. Uhlmann, Charles E. Mackel, Evgeny Deforzh, Rosalia Rabinovsky, Priscilla K. Brastianos, Hemant Varma, Rafael A. Vega, Anna M. Krichevsky

**Affiliations:** 1 Department of Neurology, Beth Israel Deaconess Medical Center, Harvard Medical School, 330 Brookline Avenue, Boston, MA 02215 USA; 2 Department of Neurosurgery, Beth Israel Deaconess Medical Center, Harvard Medical School, 110 Francis Street, Boston, MA 02215 USA; 3 Department of Neurology, Brigham and Women’s Hospital, Harvard Medical School, Boston, MA 02115 USA; 4 Department of Neurology, Massachusetts General Hospital, Harvard Medical School, Boston, MA 02114 USA; 5 Department of Pathology, Beth Israel Deaconess Medical Center, Harvard Medical School, 330 Brookline Avenue, Boston, MA 02215 USA

**Keywords:** Meningioma, miR-483-5p, IGF-2, IGF1R, Ceritinib

## Abstract

**Purpose:**

Meningioma is the most common primary central nervous system tumor often causing serious complications, and presently no medical treatment is available. The goal of this study was to discover miRNAs dysregulated in meningioma, and explore miRNA-associated pathways amenable for therapeutic interventions.

**Methods:**

Small RNA sequencing was performed on meningioma tumor samples to study grade-dependent changes in microRNA expression. Gene expression was analyzed by chromatin marks, qRT-PCR and western blot. miRNA modulation, anti-IGF-2 neutralizing antibodies, and inhibitors against IGF1R were evaluated in a tumor-derived primary cultures of meningioma cells.

**Results:**

Meningioma tumor samples showed high, grade-dependent expression of miR-483-5p, associated with high mRNA and protein expression of its host gene IGF-2. Inhibition of miR-483-5p reduced the growth of cultured meningioma cells, whereas a miR-483 mimic increased cell proliferation. Similarly, inhibition of this pathway with anti-IGF-2 neutralizing antibodies reduced meningioma cell proliferation. Small molecule tyrosine kinase inhibitor blockade of the IGF-2 receptor (IGF1R) resulted in rapid loss of viability of cultured meningioma tumor-derived cells, suggesting that autocrine IGF-2 feedback is obligatory for meningioma tumor cell survival and growth. The observed IGF1R-inhibitory IC50 for GSK1838705A and ceritinib in cell-based assays along with the available pharmacokinetics data predicted that effective drug concentration could be achieved in vivo as a new medical treatment of meningioma.

**Conclusion:**

Meningioma cell growth is critically dependent on autocrine miR-483/IGF-2 stimulation and the IGF-2 pathway provides a feasible meningioma treatment target.

**Supplementary Information:**

The online version contains supplementary material available at 10.1007/s11060-023-04264-z.

## Introduction

Meningioma is the most frequently reported primary tumor in the Central Brain Tumor Registry of the United States [1]. Presently no broadly effective medical treatment is available owing to the lack of appropriate disease models suitable for drug screening and for the study of the underlying pathology [2]. Histological grading and measurement of residual tumor by neuroimaging helps to estimate the risk of recurrence after surgery [3]. Biallelic inactivation of *NF2* is present in 40–80% of sporadic meningiomas. Other genetic alterations include mutations in *TRAF7*, *AKT1, KLF4, PIK3CA, SMO, POLR2A, SMARCB1*, and the telomerase-reverse transcriptase promoter [4–7].

Meningioma research is slowed down by the lack of practical high fidelity models. Currently used cell lines are derived from grade 2 or 3 disease, which are less common and more aggressive than grade 1 meningioma, thus these cell lines are not representative of the most common pathology. Furthermore, these cell lines accumulated additional genetic alterations during establishment and passage that are not part of meningioma pathology. Despite these drawbacks, these lines are convenient to use given the high proliferation rate and ability to grow as xenotransplants [8, 9]. We developed a primary tumor-derived meningioma culture system which recapitulates the disease with high-fidelity [10]. The presence of stromal and vascular endothelial cells in these cultures offer additional advantages, allowing for a closer recapitulation of the tumor microenvironment. The disadvantage of this model, however, is that each tumor-derived culture is unique. Furthermore, the primary cells have limited proliferative potential, not allowing for multiple assays on a single tumor derivative. Finally, this model is not practical for xenotransplantation given the slow growth rate.

Dysregulated miRNA expression is one of the characteristics of various malignancies and miRNA signatures have been established for many tumors. Moreover, miRNAs facilitating or suppressing tumor growth have been identified. Some such oncogenic or tumor suppressor miRNAs are promising biomarkers or even therapeutic targets [11–13]. However, the miRNAs driving meningioma progression, and the role of miRNA-mediated signaling in meningioma remains to be investigated. In this study, we used small RNA sequencing of meningioma samples to identify differentially expressed miRNAs by grade to better understand the cellular drivers of tumor development and progression. We found that elevated expression of miR-483-5p and miR-675-5p, two closely encoded miRNAs, is associated with meningioma grade, suggesting that their host *H19-IGF2* locus is activated. This is a complex locus containing the IGF-2 gene with its multiple promoters, intronic miR-483-5p and the *H19* gene 128 kb downstream with the intronic miR-675-5p. IGF-2 normally promotes growth during gestation, whereas postnatal activation of the *H19-IGF2* locus is oncogenic [14]. Intriguingly, tumors overexpressing IGF-2 invariably have high expression of H19, miR-483-5p and miR-675-5p as well [15]. IGF-2 normally promotes growth during gestation, whereas postnatal activation of the *H19-IGF2* locus is oncogenic [14]. There is evidence that miR-483-5p transcriptionally activates IGF-2 expression [16, 17]. Meningioma tumor cells also express a high level of IGF1R, the main receptor for IGF-2 [18]. Here, using primary meningioma cells, we demonstrate that inhibition of either miR-483-5p or IGF-2 reduce meningioma growth offering a new therapeutic strategy for these highly prevalent brain tumors.

## Materials and methods

### Cell lines and primary cultures

Tumor-derived fresh tissues utilized for meningioma primary cultures are described in Supplementary Table 1. The cultures were established as previously reported [10]. Tumor tissue was enzymatically dissociated and unsorted cells were cultured in neurobasal medium with 4% FCS. Subconfluent cultures were expanded by trypsinization and replating. After attachment, the media was changed to Neurobasal Medium with N2 and B27 for subsequent assays. The meningioma-derived C157-MN and F5 cell lines were generated and shared by Robert Martuza (Massachusetts General Hospital, Boston, MA, USA). Tumor-derived fresh-frozen tissues used for RNA sequencing and previously analyzed by genome sequencing [6] are described in Supplementary Table 2. HepG2 (RRID:CVCL 0027) was purchased from ATCC (HB-8065, ATCC, Manassas, VA). The AC007T immortalized human arachnoid cell line was generated and shared by Vijaya Ramesh (Massachusetts General Hospital, Boston, MA, USA). AC007T, C157-MN and F5 were authenticated commercially by short tandem repeat DNA profiling (Labcorp, Burlington, NC). All cell cultures were tested [19] and were mycoplasma-free.

### Meningioma small RNA sequencing

RNA was isolated from tissue sections obtained from fresh-frozen blocks, using column purification (PureLink RNA Mini, ThermoFisher Scientific, Waltham, MA, USA). Small RNA sequencing was performed at the Center for Cancer Computational Biology, Dana Farber Cancer Institute. Libraries were prepared using a multiplex small RNA kit (NEBNext, E7300, New England Biolabs, Ipswich, MA, USA) followed by column purification and size selection (AMPure XP, Beckman Coulter, Indianapolis, IN, USA). Sequencing was performed using a flow cell with 11 samples in one lane and 10 samples in the second lane (SR50, HiSeq, Illumina, San Diego, CA, USA).

### Transfection and metabolic assay

Meningioma tumor-derived cultures were transfected as previously described [10], using 0.22 nmol of oligonucleotides and 1.25 µl transfection reagent per well (Lipofectamine 2000, 11,668,027, ThermoFisher Scientific, Waltham, MA, USA). The medium was changed after six hours to Neurobasal Medium with N2 and B27 (ThermoFisher Scientific, Waltham, MA, USA). Forty-eight hours after transfection the medium was replaced with DMEM containing 10% WST-1 (11,644,807,001, Roche, Mannheim, Germany). The plates were assayed after 20 min at 450 nm using an optical reader (GloMax Multi+, Promega, Madison, WI, USA).

### Oligonucleotides and small molecule inhibitors

Hsa-miR-483-5p mimic, YM00473215-ADA, mimic control, YM00479902-ADA, inhibitors hsa-miR-675-5p, 427419-00, hsa-miR-675-3p, 427420-00, hsa-miR-483-5p, 427155-00, hsa-miR-483-3p, 427154-00, hsa-miR-204-5p, 426934-00, hsa-miR-135-5p, 426790-00, hsa-miR-10a-5p, 426651-00, hsa-miR-9-5p, 427460-00, hsa-miR-9-3p, 427461-00, Control B, 199021-00 are from Qiagen, Germantown, MD, USA. Ceritinib, S7083, linsitinib, S1091, entrectinib, S7998, GSK1904529A, S1093, picropodophyllin, S7668 were obtained from Selleck Chemicals, Houston, TX, USA; GSK1838705A, SML0995, was purchased from Millipore-Sigma, Burlington, MA, USA. The primers used for miRNA and mRNA quantification were miR-99b-5p, 4,427,975, miR-483-5p, 4,427,975, miR-675-5p, 4,427,975, IGF-2, 4,331,182, GAPDH, 4,331,182, ThermoFisher Scientific, Waltham, MA, USA.

### Analysis of IGF-2 expression

IGF-2 expression microarray data from 109 samples including 96 tumors representing the major meningioma mutation groups (*NF2*/chr22 loss, *POLR2A, KLF4/TRAF7, PI3K* mutant, and Sonic Hedgehog mutant) and 13 adult meninx tissue controls are displayed. This data is publicly available [20]. The center line of the box shows the median expression, box limits indicate the 25th and 75th percentiles, and whiskers extend to the minimum and maximum values.

### Inhibition of Akt phosphorylation

HepG2 cells were plated in 100 mm dishes and grown in DMEM with 10% FCS. Subconfluent cultures were washed three times with PBS and serum-starved for one hour in DMEM. Small molecule inhibitors were added at 100 times the IC50 concentration for 30 min. The cell-free assay IC50 values for each inhibitor are summarized in Table 1, along with the observed cell-based assay IC50 values. Human recombinant IGF-2 (292-G2, R&D Systems, Minneapolis, MN, USA) was added at a concentration of 200 ng/ml for 15 min.

**Table 1:** Summary of the half-inhibitory concentrations of the IGF1R inhibitors used. The cell-free IC50 values were provided by the drug supplier. The cell-based IC50 values were determined experimentally. The average and the range are listed from three cultures tested (MEN049, MEN051, MEN052). The peak plasma concentrations were published elsewhere [28–30]

	IGF-1R IC50	IGF-1R IC50	Peak plasma concentration
	[nM, cell-free assay]	[nM, cell culture assay]	[nM]
GSK1838705A	2	31 (28-32)	190
GSK1904529A	27	no activity	Unknown
Ceritinib	8	773 (400-960)	1500
Picropodophyllin	1	no activity	Unknown
Linsitinib	35	3500 (1540-4375)	44

### Western blot

Cultured cells and tumor samples were processed for western blot as previously described [10], except the membrane was incubated in 1 M DTT solution at room temperature for 5 min then washed with TBS-T three times before adding IGF-2 antibody. Phosphatase inhibitors (P2850, P5726, Millipore-Sigma, Burlington, MA, USA) were added to the lysis buffer for P-Akt. The antibodies used were IGF-2, 8H1, MA5-17096, 1:1000, ThermoFisher Scientific, Waltham, MA, USA, α-tubulin, Ab7291, 1:10,000, Abcam, Cambridge, UK, GAPDH, sc-47,724, 1:1000, Santa Cruz Biotechnology, Dallas, Texas, USA, Akt, 2938, P-Akt, 4058, 1:1000, secondary antibodies 7074 and 7076 S, 1:10,000, Cell Signaling Technology, Danvers, MA.

### Inhibition of meningioma cell growth

Primary cells were seeded in 96-well plates in Neurobasal Media with N2, B27 and 10% FCS at 10–50% confluence. For neutralizing antibody treatment, the cells were washed with DMEM twice then with Neurobasal Media once, then 100 µl of Neurobasal Media was added to each well. The antibodies were washed to remove sodium azide by adding 0.5 ml protein G magnetic particles (Dynabeads, 10003D, ThermoFisher Scientific, Waltham, MA, USA) in PBS, washed once with PBS and eluted with 90 µl 0.1 M citric acid pH 2.0, neutralized with 10 µl 1 M TRIS base, and the concentration was adjusted to 400 ng per µl. Antibody (400 ng) was added to each well. The cells were washed twice with PBS, then assayed 24 h later. For small molecule inhibitor assays, the compounds were added directly to the media 24 h after plating, with serial dilution starting at 500 times the IC50 concentration. After 72 h, cells were washed with PBS twice, the media was replaced with DMEM plus 10% WST-1 and assayed after 20 min at 450 nm.

### Chromatin mark analysis

ChIP-Seq data for H3K27Ac, H3K4Me3, H3K9Ac, H3K27Me3 and H3K9Me3 chromatin marks covering the *H19-IGF2* locus in meningioma were retrieved from a public repository (https://dataverse.tdl.org) and signal tracks were assembled using an open source application [21].

### Real-time PCR

Total RNA (3 ng) was used for reverse transcription and real-time PCR assays (Superscript III First-Strand Synthesis, 11,752,250, Taqman Universal Master Mix II, 4,440,040, Quantstudio 7 Flex, ThermoFisher Scientific, Waltham, MA, USA). Cycle threshold values for miR-483-5p and miR-675-5p were normalized to those of miR-99b-5p which was uniformly and highly expressed in all meningioma tumor samples. The IGF-2 mRNA expression was normalized to that of GAPDH.

### Data analysis

Small RNA quantification was performed using an open-source algorithm [22]. Unpaired two-tailed t-tests and one-way analysis of variance tests were performed using commercial software (GraphPad Prism 8.3.0). Linear regression was used for the expression analysis. The standard error of the mean is shown for each category In the bar graphs. A heatmap with hierarchical clustering was generated using open-source software (heatmap.2, gplots, R 3.1.3).

## Results

### Mir-483-5p and mir-675-5p are overexpressed in meningiomas

**Fig. 1:**
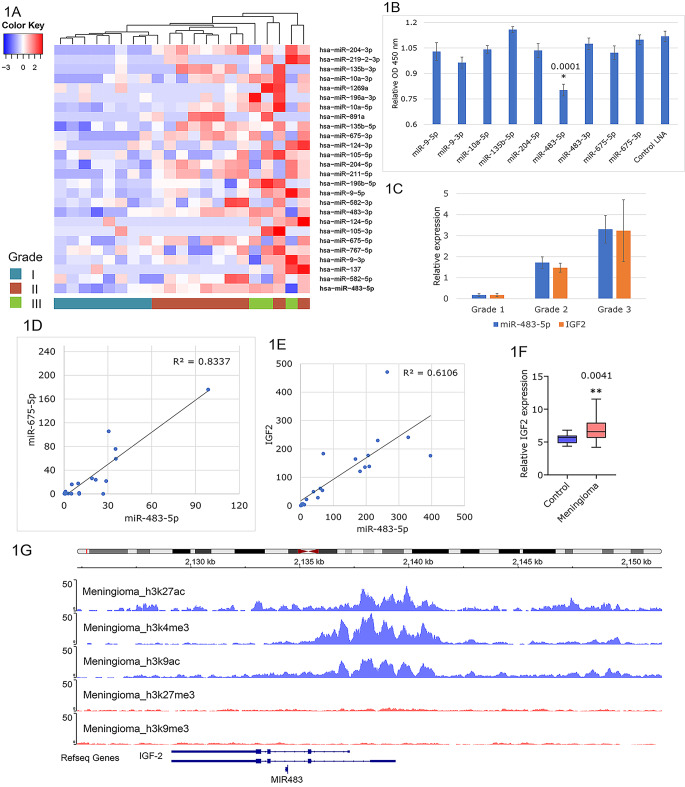
Identification of miR-483-5p and IGF-2 upregulation in meningiomas. **A**, Heatmap representation of differentially expressed miRNAs, based on sequencing, in the meningioma tumor samples by grade. Cluster analysis is shown at the top and the grade is indicated at the bottom. Z-scores are defined on the color key. **B**, Screening of the candidate miRNAs using oligonucleotide inhibitors in F5 cells showed reduced viability with miR-483-5p inhibition. **C**, IGF-2 mRNA and miR-483-5p expression as determined by RT-PCR, were compared as a function of meningioma grade (n = 21). **D**, miR-483-5p and miR-675-5p expression levels correlated in the meningioma tumor samples (n = 21). **E**, IGF-2 mRNA and miR-483-5p expression were also directly correlated. **F**, IGF-2 was overexpressed in meningioma samples (n = 96) compared with control samples (n = 13). Center lines indicate the median, box limits indicate the 25th and 75th percentiles, and whiskers extend to the minimum and maximum values. The two-tailed unpaired t-test p-value is shown at the top. **G**, The H19-IGF2 locus is epigenetically active in meningiomas. Active chromatin marks, H3K27Ac, H3K4Me3, and H3K9Ac are shown in blue and are enriched. In contrast, silent chromatin marks, H3K27Me3, and H3K9Me3 are shown in red and are mostly absent

Meningioma originates from arachnoid cap cells of the sparse middle layer of the brain linings. Since this normal tissue as control is unavailable, we investigated miRNA profiles of meningioma using resected tumor samples of different grades. miRNA counts from 21 fresh-frozen meningioma tumor samples were ranked according to their expression levels and differential expression between the tumor grades. Of the top 26 miRNAs differentially expressed in grades 1 and 2 (Fig. 1a), several were 3p/5p counterparts derived from the same pre-miRNAs. miR-483-5p/3p and miR-675-5p are located merely 137 Kb apart and are co-expressed upon locus activation [15]. Nine miRNAs were selected for further testing based on their established growth-regulatory functions in other tumor types [23–26] (Fig. 1b). The metabolic effects of targeting each of these miRNAs with specific oligonucleotide inhibitors were first assayed in F5 meningioma cells. Of the nine differentially expressed miRNAs, specific inhibition of miR-483-5p resulted in significantly reduced metabolic activity, whereas inhibitors of other miRNA had no effect (Fig. 1b).

The levels of miR-483-5p in the meningioma samples increased with grade (Fig. 1c) and there was a high correlation between miR-483-5p and miR-675-5p expression (Fig. 1d). Furthermore, miR-483-5p levels correlated closely with expression of its host gene IGF2 (Fig. 1e), suggesting the activation of the entire *H19-IGF2* locus. Analysis of an additional public dataset, with 96 meningioma samples and 13 normal meninx controls confirmed that IGF-2 mRNA is upregulated in meningioma compared to controls (Fig. 1f). Furthermore, chromatin mark analysis of the *H19-IGF2* locus in meningiomas revealed epigenetic activation. Specifically, active chromatin marks H3K27Ac, H3K4Me3, and H3K9Ac were enriched, while silent marks H3K27Me3 and H3K9Me3 were absent (Fig. 1g). Altogether, these results suggested activation of the *H19-IGF2* locus in meningioma.

### IGF-2 is overexpressed in meningiomas

**Fig. 2:**
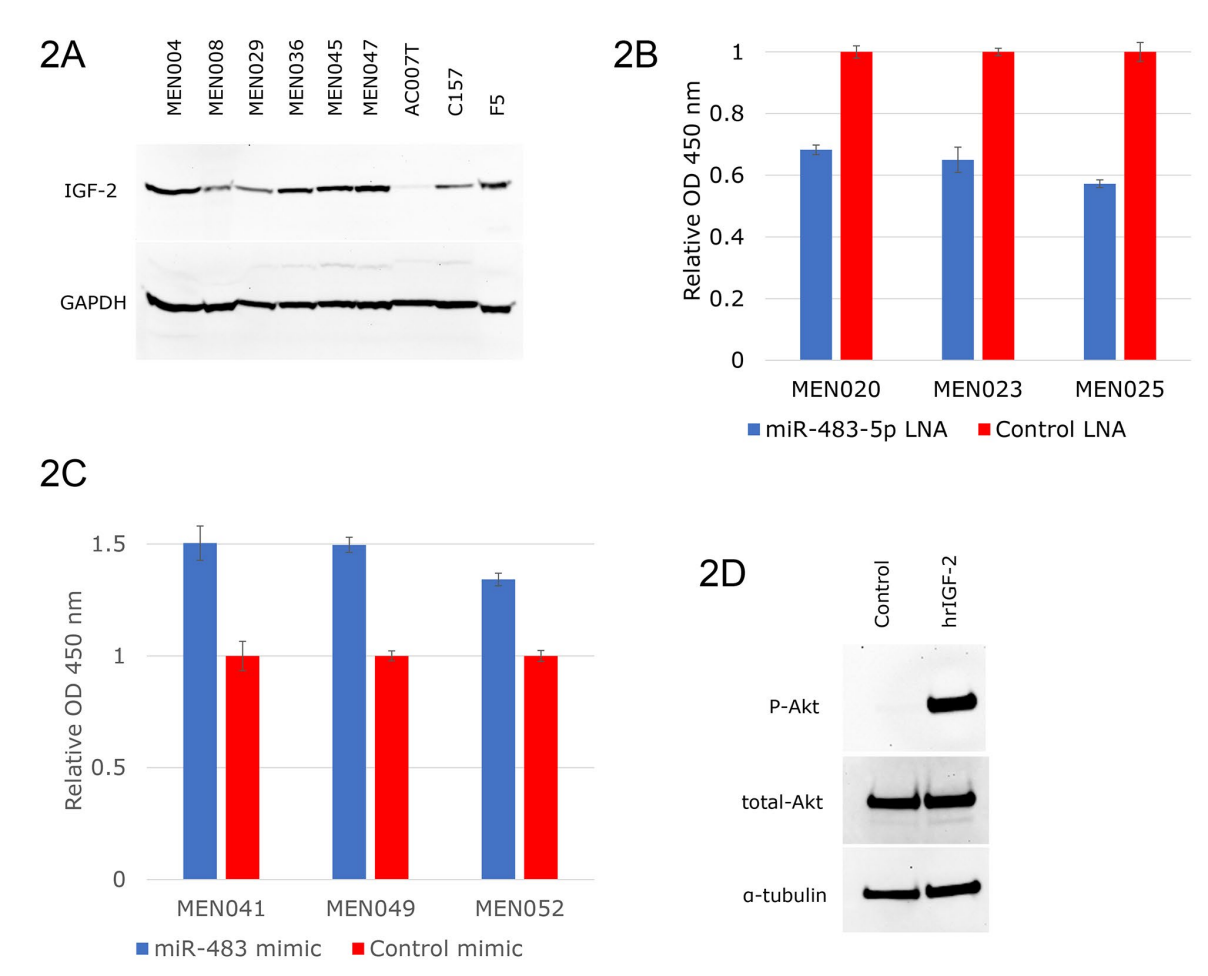
Meningioma tumors exhibit IGF-2 protein expression and growth dependence on miR-483-5p and IGF-2. **A**, Western blot of meningioma tumor samples and cell cultures showing IGF-2 expression. **B**, WST-1 metabolic activity assay of cultured tumor-derived meningioma cells transfected with miR-483-5p inhibitor and assayed 48 h post-transfection. **C**, WST-1 metabolic activity of cultured tumor-derived meningioma cells transfected with miR-483-5p mimic. **D**, Tumor-derived meningioma cells undergo mitogenic activation upon exposure to recombinant IGF-2.

Since miR-483-5p, miR-675-5p, and IGF-2 mRNA appeared to be closely linked and upregulated in meningioma samples, we tested additional meningioma tumor samples (Supplementary Table 1) for IGF-2 protein expression. Western blot analysis revealed that most meningiomas expressed high levels of IGF-2. Here, a subset of samples are shown (Fig. 2a), other tumor samples tested showed similar results (data not shown). Established meningioma cell lines C157-MN and F5 also express high level IGF-2 although lower than the tumor samples. The immortalized arachnoid (non-meningioma) cell line AC007T showed low level of IGF-2 expression (Fig. 2a). Similarly, the miR-483-5p expression is present in these cell lines, although weaker than in the tumor samples (data not shown).

### Mir-483-5p is oncogenic cultured meningioma cells

To investigate the function of miR-483-5p/IGF-2 circuit in meningioma, we first assessed effects of miR-483-5p modulation on meningioma growth. Inhibition of miR-483-5p in the cultured meningioma-derived cells (MEN020, MEN023, and MEN025) strongly inhibited metabolic activity, suggesting a critical role for miR-483-5p in maintaining the meningioma tumor state (Fig. 2b). Conversely, the transient elevation of miR-483-5p with a transfected mimic showed increased metabolic activity (Fig. 2c). It has been previously reported that miR-483-5p activates the expression of IGF-2 at the transcriptional level [14]. We hypothesized that the effect of miR-483-5p on cell viability and growth is mediated by the elevated IGF-2. To examine whether IGF-2 is a mitogen in meningiomas, recombinant human IGF-2 was added to the media of cultured meningioma-derived cells after serum starvation. Western blot analysis showed robust Akt phosphorylation after IGF-2 exposure, suggesting that IGF-2 is capable of activating a mitogenic pathway and thus stimulating meningioma growth (Fig. 2d).

### IGF-2 pathway inhibition in meningioma-derived cultured cells

**Fig. 3:**
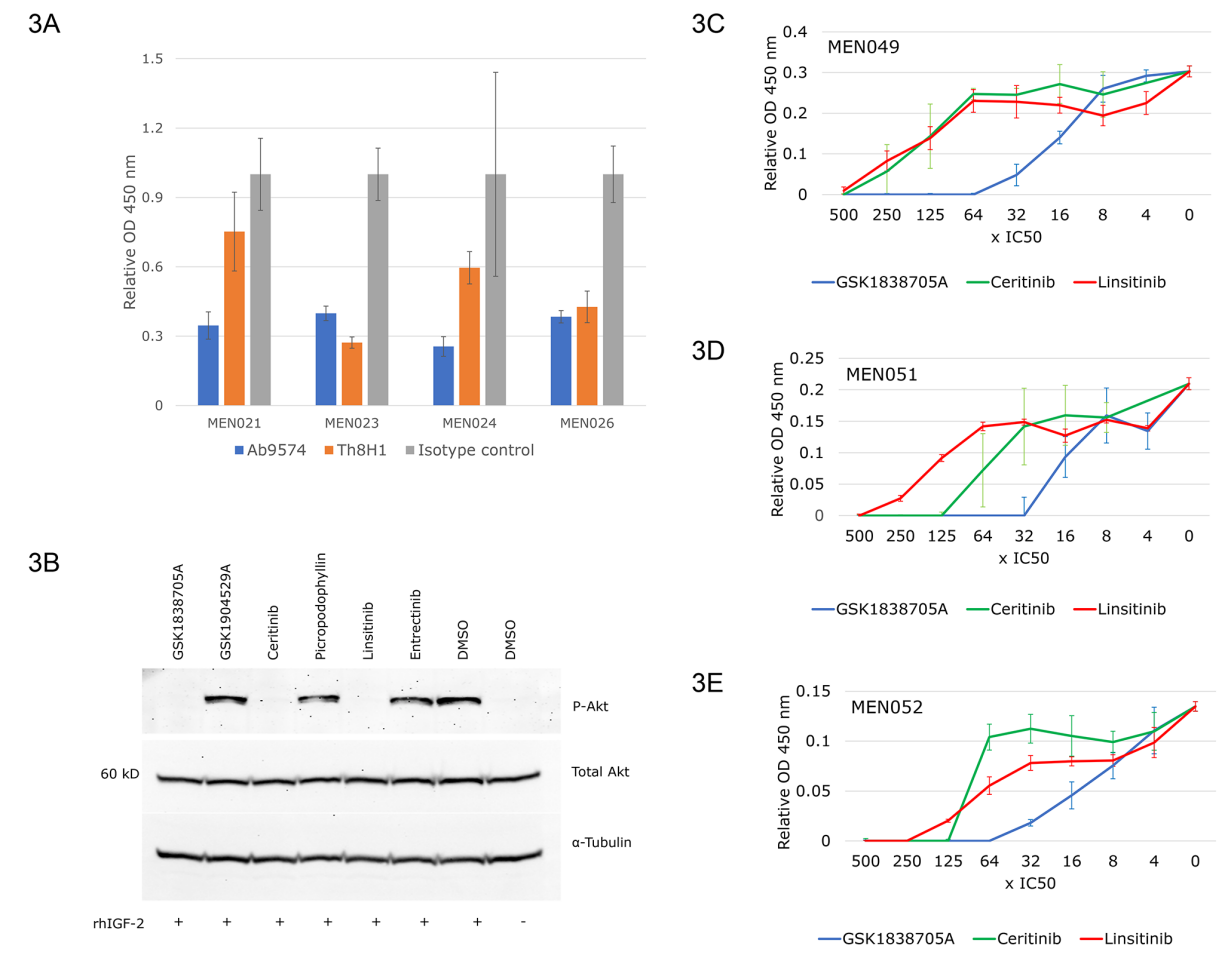
The cytokine IGF-2 and its receptor IGF1R are both essential for meningioma tumor growth. **A**, Anti-IGF2 neutralizing antibodies reduce the metabolic activity of cultured tumor-derived meningioma cells, as assayed by WST-1. **B**, Western blot analysis validating the efficacy of specific IGF1R small-molecule receptor tyrosine kinase inhibitors to block IGF-2-induced Akt phosphorylation in HepG2 cells. The ALK inhibitor entrectinib is used as a negative control. **C-E**, Metabolic assay of cultured tumor-derived meningioma cells was performed 72 h after treatment with IGF1R small molecule receptor tyrosine kinase inhibitors. The inhibitor concentrations used are expressed in multiples of the published cell-free IC50 values for each drug

To further investigate the effects of blocking IGF-2 signaling, we tested various IGF-2 pathway inhibitors. Adding two distinct anti-IGF-2 neutralizing antibodies to the culture media resulted in reduced cell growth in diverse primary meningioma cultures (Fig. 3a). We then asked whether small molecule inhibitors of IGF-2 signaling would have similar effects. First, to validate the activity of six candidate small molecules, we tested their ability to block IGF-2-mediated phosphorylation of Akt. Recombinant human IGF-2 was added to HepG2 cells treated with putative inhibitors of IGF1R (Table 1), the main IGF-2 receptor, and Akt phosphorylation was assayed by western blotting (Fig. 3b). Entrectinib, an ALK/ROS/Trk inhibitor with no IGF1R inhibitory activity was used as the negative control. GSK1904529A and picropodophyllin at 100 times the cell-free IC50 did not block IGF-2-mediated Akt-phosphorylation (Fig. 3d). In contrast, GSK1838705A, ceritinib, and linsitinib blocked IGF-2-mediated Akt-phosphorylation, indicating their strong IGF-2-inhibitory properties. In parallel, these three drugs caused growth inhibition of all the tumor-derived cultured meningioma cells tested. Three examples are shown, MEN049, MEN051 and MEN052 (Fig. 3c and d, and 3e). Similarly to other small molecule receptor tyrosine kinase inhibitors, the growth-inhibitory IC50 values in the cell-based assay were higher then those of the cell-free assay (Table 1). Consistent with their lack of IGF-2 pathway inhibitory activity, GSK1904529A and picropodophyllin did not affect cell growth (data not shown).

## Discussion

miRNA sequencing of meningioma samples identified a set of highly expressed miRNAs that differentiated grade 1 and grade 2 meningiomas. A study of signaling pathways and miRNA in 16 grade 1 and 16 grade 2 meningioma samples found a similar pattern of miRNA expression including miR-483-5p upregulation [20], however, the functional activity of miR-483-5p and IGF-2 was not explored by these authors.

Our study supports the feasibility of using miRNA signatures for the discovery of signaling pathway alterations in oncology research. The identification of miR-483-5p led us to investigate the epigenetically activated host gene that expresses IGF-2. Although IGF-2 upregulation has been previously reported in meningiomas [27], along with *H19-IGF2* locus imprinting [16] and overexpression of miR-483-5p [20], comprehensive IGF-2 expression analysis has been hampered by technical difficulties using formaldehyde-fixed paraffin-embedded samples. In contrast, western blot analysis of fresh-frozen tumor samples reproducibly showed high IGF-2 protein expression in all meningiomas studied. IGF-2 is a powerful mitogen for meningioma cells as demonstrated by Akt phosphorylation following addition of recombinant IGF-2. Whereas inhibition of either miR-483 or IGF-2 may provide therapeutic strategy for meningioma, miRNA-targeting with oligonucleotide drugs have not yet been clinically established for brain tumors. In contrast, small molecule or antibody inhibitors of IGF-2 signaling could be easily implemented in clinical trials.

Among the orally available IGF-1R inhibitors, GSK1838705A, ceritinib, and linsitinib strongly inhibited meningioma cell growth in our in vitro model. IGF-2-mediated Akt phosphorylation was also strongly blocked by GSK1838705A, ceritinib and linsitinib suggesting that the mechanism by which these drugs inhibit meningioma cell growth is mainly via IGF1R blockade. IGF1R blockade with linsitinib was previously found to be ineffective in adrenocortical carcinoma [28]. The oral dose used in one study, 150 mg twice daily, was well tolerated with few hyperglycemic adverse effects. However, the measured peak plasma concentration of 44 nM is well below the observed IC50 of 3500 nM in our cell-based assay, providing a plausible explanation for the lack of in vivo efficacy. GSK1838705A has not yet been tested in US-registered clinical trials. Ceritinib has been tested in clinical trials [29], and shown efficacy in malignant tumors with ALK rearrangement. However, its effectiveness in that setting is due to its strong ALK inhibitor activity, rather than the modest IGF1R inhibition.

Despite the lack of efficacy observed in high grade cancers, our data suggests that IGF1R blockade has promise in meningioma treatment, given meningioma’s apparent high dependence on the IGF-2 pathway. Owing to the lower proliferation rate, and the resulting low mutation rate in meningiomas compared to high-grade malignancies, escape mutations are improbable. Additionally, the lack of a blood-brain barrier between the systemic circulation and meningioma tumor cells may allow for an effective drug concentration at the target. GSK1838705A and ceritinib showed strong inhibition of cultured meningioma tumor-derived cells, with observed IC50 values of 31 nM and 773 nM, while peak plasma concentrations were 190 nM and 1500 nM respectively. Consequently, both drugs are predicted to be therapeutically effective (Table 1), although for GSK1838705A only mouse pharmacokinetics data is available presently [30].

The therapeutic use of anti-IGF-2 neutralizing antibodies may offer better tolerability by avoiding hyperglycemia, a common side effect of small molecule IGF1R inhibitors, due to cross-reaction with the Insulin Receptor. Indeed, a clinical trial using an anti-IGF1/2 antibody has shown excellent tolerability [31]. However, in our model, small molecule tyrosine kinase inhibitors showed much stronger efficacy, resulting in a complete loss of viability of meningioma cells, while neutralizing antibodies had only a modest effect on the metabolic rate. A possible explanation is that most circulating IGF-2 is sequestered by IGFBP-3 and thus inaccessible for neutralization. IGF-2 is released from IGFBP-3 in the extravascular space, where its neutralization is in turn hampered by poor penetration of the antibody [32]. The high cost of recombinant antibodies and the inconvenience of parenteral administration further limit this approach. Taken together, our data indicate that IGF1R inhibitors, including GSK1838705A and ceritinib, should be evaluated in mouse meningioma models and in clinical trials.

This study has several limitations. First, the analysis of miRNAs differentially expressed between grade 1 and grade 2 tumors, rather than normal control cells is an indirect approach to probe miRNA dysregulation. The cell of origin of meningiomas is thought to be arachnoid cap cells, and ideally this should be used as control. However, normal arachnoid cells are not present in the surplus tumor specimens used for research. The second limitation is that each tumor-derived culture is unique, requiring testing on multiple cultures. Primary cells have a limited proliferative potential, not allowing for all desired assays to be performed on a single primary culture. Finally, this model is not practical for xenotransplantation given the low growth rate. These problems, however, may be offset by the high fidelity of the model. Although results from individual tumor-derived cultures exhibit some variation, a high level of significance can be achieved by assaying several independent cultures in parallel.

In summary, we investigated the miRNA landscape of meningioma, and found miR-483-5p overexpression via epigenetic activation of the H19-IGF2 locus. Most tumor samples tested showed high IGF-2 protein expression. Inhibition of the miR-483/IGF-2 pathway with miR-483 oligonucleotide inhibitor, anti-IGF-2 neutralizing antibodies, and IGF1R small molecule tyrosine kinase inhibitors caused rapid loss of viability of cultured meningioma tumor-derived cells. Meningioma cell growth appears to be critically dependent on autocrine IGF-2 stimulation and the IGF-2 pathway provides a possible meningioma treatment target. Of the available IGF1R inhibitors, GSK1838705A and ceritinib are predicted to be effective in vivo, based on data from cell-based assays and pharmacokinetic tests.

## Electronic supplementary material

Below is the link to the electronic supplementary material.

Supplementary Material 1

Supplementary Material 2

## Data Availability

The data generated during the current study are available from the corresponding author on request.

## Abbreviations

AKT1: RAC-alpha serine/threonine-protein kinase
ALK: Anaplastic lymphoma kinase
DMEM: Dulbecco’s modified eagle medium
DTT: Dithiothreitol
FCS: Fetal calf serum
GAPDH: Glyceraldehyde-3-phosphate dehydrogenase
H3: Histone H3
IC50: 50% inhibitory concentration
IGF1R: Insulin-like growth factor receptor
IGF-2: Insulin-like growth factor 2 (protein)
IGF2: Insulin-like growth factor 2 (gene)
KLF4: Kruppel-like factor 4
miR: microRNA
NF2: Neurofibromatosis type 2
PI3K: Phosphatidylinositol 3-kinase
PIK3CA: Phosphatidylinositol-4,5-bisphosphate 3-kinase, catalytic subunit alpha
POLR2A: DNA-directed RNA polymerase II subunit
qRT-PCR: Quantitative real-time polymerase chain reaction
ROS: Tyrosine-protein kinase from ornithon sarcoma virus
SMARCB1: Switch/sucrose non-fermentable-related matrix-associated actin-dependent regulator of chromatin subfamily B member 1
SMO: Smoothened
TRAF7: TNF receptor associated factor 7
Trk: Tropomyosin receptor kinase receptor
